# The Association Between Dissemination and Characteristics of Pro-/Anti-COVID-19 Vaccine Messages on Twitter: Application of the Elaboration Likelihood Model

**DOI:** 10.2196/37077

**Published:** 2022-06-27

**Authors:** Vipin Saini, Li-Lin Liang, Yu-Chen Yang, Huong Mai Le, Chun-Ying Wu

**Affiliations:** 1 Department of Information Management College of Management National Sun Yet-sen University Kaohsiung Taiwan; 2 Institute of Public Health College of Medicine National Yang Ming Chiao Tung University Taipei Taiwan; 3 Department of Business Management College of Management National Sun Yat-sen University Kaohsiung Taiwan; 4 Research Center for Epidemic Prevention National Yang Ming Chiao Tung University Taipei Taiwan; 5 Health Innovation Center National Yang Ming Chiao Tung University Taipei Taiwan; 6 Institute of Biomedical Informatics College of Medicine National Yang Ming Chiao Tung University Taipei Taiwan

**Keywords:** COVID-19, Twitter, provaccine, antivaccine, elaboration likelihood model, infodemiology, dissemination, content analysis, emotional valence, social media

## Abstract

**Background:**

Messages on one’s stance toward vaccination on microblogging sites may affect the reader’s decision on whether to receive a vaccine. Understanding the dissemination of provaccine and antivaccine messages relating to COVID-19 on social media is crucial; however, studies on this topic have remained limited.

**Objective:**

This study applies the elaboration likelihood model (ELM) to explore the characteristics of vaccine stance messages that may appeal to Twitter users. First, we examined the associations between the characteristics of vaccine stance tweets and the likelihood and number of retweets. Second, we identified the relative importance of the central and peripheral routes in decision-making on sharing a message.

**Methods:**

English-language tweets from the United States that contained provaccine and antivaccine hashtags (N=150,338) were analyzed between April 26 and August 26, 2021. Logistic and generalized negative binomial regressions were conducted to predict retweet outcomes. The content-related central-route predictors were measured using the numbers of hashtags and mentions, emotional valence, emotional intensity, and concreteness. The content-unrelated peripheral-route predictors were measured using the numbers of likes and followers and whether the source was a verified user.

**Results:**

Content-related characteristics played a prominent role in shaping decisions regarding whether to retweet antivaccine messages. Particularly, positive valence (incidence rate ratio [IRR]=1.32, *P*=.03) and concreteness (odds ratio [OR]=1.17, *P*=.01) were associated with higher numbers and likelihood of retweets of antivaccine messages, respectively; emotional intensity (subjectivity) was associated with fewer retweets of antivaccine messages (OR=0.78, *P*=.03; IRR=0.80, *P*=.04). However, these factors had either no or only small effects on the sharing of provaccine tweets. Retweets of provaccine messages were primarily determined by content-unrelated characteristics, such as the numbers of likes (OR=2.55, IRR=2.24, *P*<.001) and followers (OR=1.31, IRR=1.28, *P*<.001).

**Conclusions:**

The dissemination of antivaccine messages is associated with both content-related and content-unrelated characteristics. By contrast, the dissemination of provaccine messages is primarily driven by content-unrelated characteristics. These findings signify the importance of leveraging the peripheral route to promote the dissemination of provaccine messages. Because antivaccine tweets with positive emotions, objective content, and concrete words are more likely to be disseminated, policymakers should pay attention to antivaccine messages with such characteristics.

## Introduction

### Background

Vaccination against COVID-19 has been promoted by governments as a key strategy to prevent infections and fatalities. The wide spread of the highly contagious omicron variant has made vaccination coverage more imperative than ever. However, an overabundance of information has prevented people from protecting themselves against COVID-19 [1]. Scholars have discovered that people are easily influenced by vaccine-related opinion pieces published on microblogging sites. For example, vaccine hesitancy is closely related to antivaccination campaigns on social media [2,3]. Therefore, understanding the dissemination of provaccine and antivaccine messages on social media websites is crucial. The World Health Organization has called for a greater focus on infodemiology, the area of science research dedicated to understanding the distribution of information through electronic mediums [4-6]. This study examined what characteristics of vaccine stance messages are likely to result in dissemination and whether those characteristics differ between provaccine and antivaccine messages. Answers to these questions will help governments proactively engage in disseminating provaccine messages and identify potentially influential antivaccine messages.

We selected Twitter as the data source because it is the most popular microblogging site, with 397 million active global users as of January 2022 [7]. Microblogging sites have proven their effectiveness in public information adoption and decision-making when used to promote a government vaccination policy [8]. Twitter allows users to retweet another user’s text to disseminate information among their followers, thus enabling widespread information diffusion.

Prior to the COVID-19 pandemic, researchers have used Twitter data to examine public opinion on vaccinations through text analysis, image analysis, topic modeling, and community detection [9,10]. More recently, studies have analyzed the sentiments, opinions, topics, and persuasion techniques related to COVID-19 vaccination on Twitter [11-14]. Furthermore, much effort has been devoted to identifying the determinants of attitudes toward COVID-19 vaccines [15-18], the origin of vaccine misinformation, and its negative effect on vaccine acceptance [19]. One paper by Germani and Biller-Andorno [20] reported that compared with provaxxers, antivaxxers tweet less but are more engaged in discussions (through replies or retweets) on Twitter.

Another line of the literature focused on persuasive message appeals, including logos (fact/logic of the argument), pathos (emotion of the argument), and ethos (credibility of the author) [21]. Those rhetoric appeals have been applied to political campaigns, health issues, fund raising, promotion of technological products, and vaccination intake [22-26]. In the *Gazette* of Australia, logos appeal has been widely utilized for vaccination strategy [26]. Utilization of pathos on antivaccine websites has been found to provide the functionality of social interactivity [27]. During the COVID-19 pandemic, New York City Department of Health and Mental Hygiene and the official Twitter account of the US government have extensively utilized rhetoric appeals for vaccine communication and to promote COVID-19 vaccination [28,29].

The existing literature suggests that research on the dissemination of provaccine and antivaccine messages during the COVID-19 pandemic has remained limited. This study applied a theoretical framework called the elaboration likelihood model (ELM) to explore message characteristics that may appeal to Twitter users. Specifically, the aims are (1) to examine the associations between message characteristics and the likelihood and number of retweets and (2) to identify the relative importance of the central and peripheral routes in decision-making on sharing a message. Because vaccine discourse on social media is polarized between groups of provaccine and antivaccine communities [30], and since provaxxers and antivaxxers hardly interact with each other on Twitter [31], we conjectured that provaccine messages were predominately shared by provaxxers and antivaccine messages predominately shared by antivaxxers. As a result, we used a common set of message characteristics and tested them separately on provaccine and antivaccine messages. We then explored the role of each route in 2 different groups and compared whether the decision-making on retweets is the same for provaxxers and antivaxxers. To the best of our knowledge, this study is the first to examine the association between the dissemination and characteristics of COVID-19 vaccine stance tweets. The results will facilitate the design of effective messages by scientists, clinicians, and policymakers to promote vaccination.

### Theoretical Framework: The Elaboration Likelihood Model

The ELM was developed by Petty and Cacioppo in 1986 [32] and is 1 of the most popular persuasion models in consumer research and social psychology. The ELM proposes that attitude changes and consequent behavior changes among individuals may be caused by 2 processing approaches: the central route and the peripheral route. The central route requires an individual to think deeply about relevant arguments in a message and reflect on the relative merits and relevance of those arguments before developing an informed decision about the target behavior. In the context of decisions to retweet on Twitter, such arguments refer to the message content, such as information richness, argument sentiment, and concreteness, of the tweet. The peripheral route, however, involves less cognitive effort. A message is accepted or rejected without any critical thinking or conscious thought. Recipients simply rely on general criteria or content-unrelated characteristics, such as the information source, to make quick decisions [33]. In the context of making a decision to retweet, such cues include the number of likes received by the tweet and whether the tweet was posted by a verified user. The ELM predicts that decisions made through central-route processing will be more difficult to alter than those formed through peripheral-route processing.

The ELM has been adopted to study the effects of persuasive communication on attitude and behavioral changes with respect to online reviews [34], health information [35], and false reviews [36]. Drawing on the ELM, Guo et al [33] investigated patients’ continual usage intentions of mobile health services and Ju and Zhang [37] investigated the factors influencing patients’ continual use of web-based diagnosis and treatment. The ELM has also been applied to explain users’ decisions to share online reviews of consumer products [38] and information on social networking sites [39]. In the field of health communication, the ELM model has helped understand the effectiveness of tobacco package warning labels [40] and designing of peripheral messages to prevent drunkorexia [41]. Despite the various empirical studies, applications of the ELM for dissemination of COVID-19 vaccine stance messages are still limited.

Other researchers have explored the effects of message content on users’ retweeting decisions without applying the ELM. Their findings have revealed the impact of argument sentiment [42,43] and hashtags [44]. Studies that did not apply the ELM and focused on content-unrelated factors have also reported positive results. Source trustworthiness, source attractiveness, and favorite counts [45] affect retweeting decisions.

In practice, to explore the central route, this study used a natural language processing (NLP) technique to construct content-related variables. Content analysis was useful for this study because it exploited timely and real-world messages collected from Twitter and allowed us to identify the actual response (retweet decisions) to specific content. Furthermore, the use of algorithmic content analysis in this study helped the analysis of big data from online discourse faster compared to traditional content analysis methods (where researchers need to formulate a coding scheme and train coders to analyze the text manually) and at scale [46]. Alternatively, experiment and survey methods can be used to discover message characteristics that appeal to users. However, concerns have been raised that convenient sampling widely used by those methods could result in sample selection bias and that the survey methodology captures self-reported behavior rather than the actual behavior/response [47].

This study is original in a number of ways. We extended the scope of the ELM to vaccine communication and clarified the relative importance of 2 psychological routes in sharing pro- and antivaccine messages. We discovered that both the central and the peripheral route play key roles in the decision-making on whether to share an antivaccine message, whereas dissemination of a provaccine message was mostly determined by the peripheral route. These findings are useful for devising effective messages to promote COVID-19 vaccination and to reach out to different communities on social media. Furthermore, we included a new variable in the central route, called concreteness, that has not been explicitly considered by ELM studies before. We borrowed the concreteness construct from construal level theory (CLT) [48], which states that concrete words help individuals understand psychological proximity to the respective object or event. Originally, CLT was developed to explain how people think about an event at a concrete or abstract level [49,50]. CLT studies have demonstrated the ability of natural language to prime concrete or abstract mindsets [51,52]—association between lexical concreteness and psychological proximity [53]. By incorporating concreteness, we have not only enriched the ELM but also extended applications of CLT to vaccine stance message dissemination.

## Methods

### The Elaboration Likelihood Model

**Figure 1:**
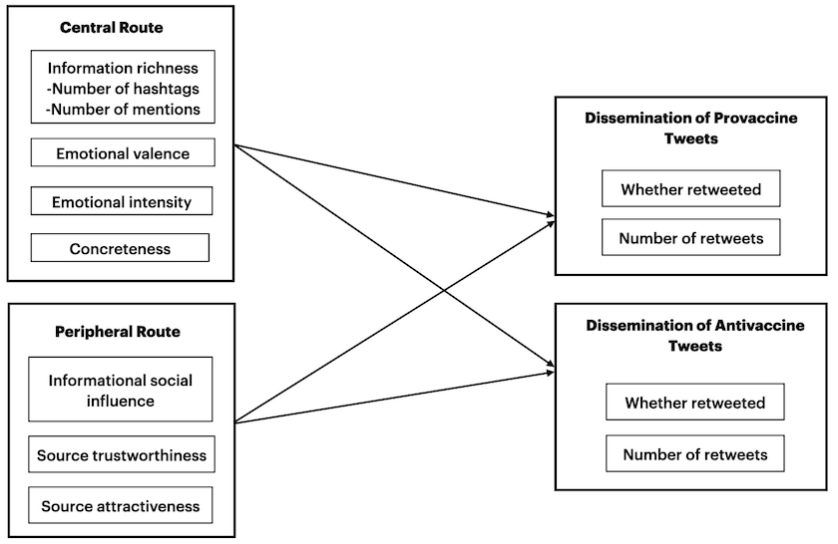
The ELM: central and peripheral routes for disseminating pro- and antivaccine tweets. ELM: elaboration likelihood model.

Our empirical analysis focused on the 2 routes of the ELM, as presented in Figure 1. We expected that when users processed information through the central route, message content would be a key predictor of dissemination, whereas when users processed information through a peripheral route, content-unrelated characteristics would be more important predictors. The central route is composed of variables for information richness, argument sentiment (emotional valence and emotional intensity), and concreteness. The peripheral route is composed of variables for informational social influence, source trustworthiness, and source attractiveness.

### Study Design, Outcome Variables, and Data Collection

To investigate retweeting behavior, a cross-sectional study design was applied to United States data. The outcome variables were (1) whether a provaccine or antivaccine tweet (collectively termed “vaccine stance tweets”) was retweeted and (2) the number of times a vaccine stance tweet was retweeted.

**Figure 2:**
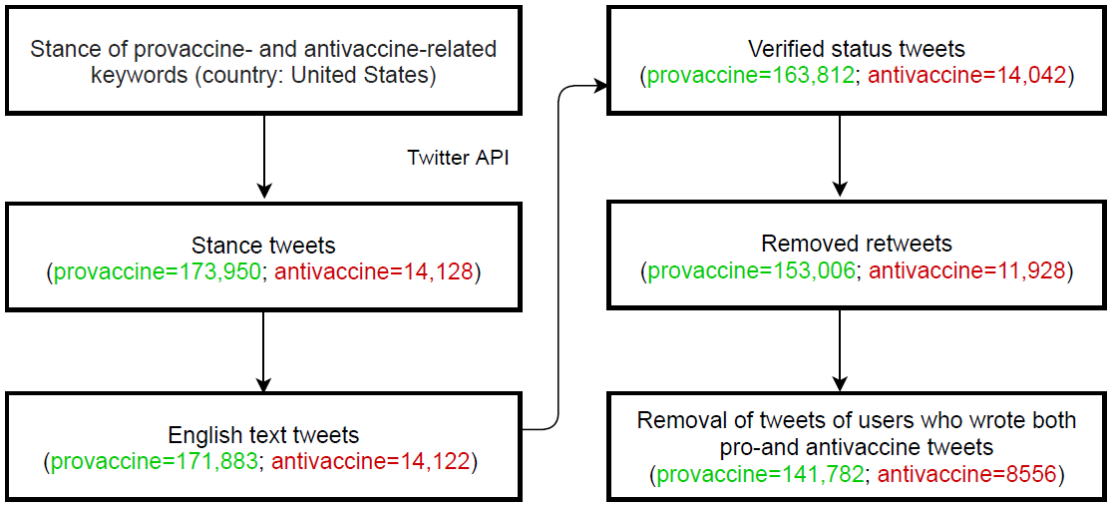
Data collection for provaccine and antivaccine tweets. This flowchart illustrates the data collection and cleaning of the final data set of vaccine stance tweets from the United States. We filtered out retweets and retained tweets from original users who had a consistent vaccine stance throughout the study periods. The green color refers to the number of provaccine tweets, and the red color refers to antivaccine tweets that remained in each step. API: application programming interface.

We used the R library (R Core Team and the R Foundation for Statistical Computing) package *rtweet* [54] to access the Twitter application programming interface (API) service to collect provaccine- and antivaccine-related tweets between April 26 and August 26, 2021. We excluded non-English tweets and tweets with a geolocation outside the United States. The provaccine search term hashtags were as follows: #GetVaccinated, #GetVaxxex, #Immunization, #Jab, #Vaccinate, #Vaccinated, #VaccinateNY, #Vaccinesafety, #vaccineswork, and #vaxxed. The following terms were used to target antivaccine tweets: #antivaxx, #antivaxxer, #naturalimmunity, #novaccinepassports, #vaccinefailure, #vaccineinjury, #vaccinemurder, #vaccinesarepoison, #vaccinedontwork, and #vaccinekill. Additionally, we looked into user IDs associated with individual tweets and excluded users who tweeted both pro- and antivaccine messages. This reduced approximately 8.8% of vaccine stance tweets identified in the original data set. The inclusion of only users whose vaccine stances remained consistent during the study period ensured that the tweets analyzed conveyed a clear stance. The final sample was composed of 141,782 provaccine and 8556 antivaccine tweets posted by 57,067 and 4308 distinct users (authors), respectively. The flowchart of Twitter data collection is presented in Figure 2.

### Predictors: Central Route

#### Information Richness

We operationalized the information richness of a tweet by using 2 measures: the number of hashtags and mentions. A hashtag is a word beginning with the # symbol, which is added to posts to aggregate messages of the same topic. A mention references another user in a microblog with the @ symbol and represents an active user interaction [55]. In the literature, the number of mentions is operationalized as a subdimension of information richness [56], and we adopted a similar method in this work.

#### Emotional Valence and Emotional Intensity

In psychology, emotional valence indicates the emotional value expressed on a continuum from unpleasant to pleasant or from negative to positive [57]. Emotional intensity is the expression of emotion in content, indicating the level of subjectivity from no emotion (objective) to highly emotional [58]. We operationalized these 2 dimensions of argument strength [43] by using TextBlob [59,60], which generated scores for these dimensions. The values of emotional valence range from −1 to 1, where −1 is extremely negative, 1 is extremely positive, and 0 is neutral. The emotional intensity values range from 0 to 1, where 0 is highly objective and 1 is highly subjective. Multimedia Appendix 1 provides examples of emotional valence and emotional intensity. For example, tweets with positive emotions/valance often contained positive words, such as “natural,” “granted,” “better,” “fine,” “good,” and “healthy.” In contrast, tweets with negative valence used negative words, including “bad,” “evil,” “terrible,” “criminal,” “sick,” “illegal,” and “painful.” TextBlob assigns individual scores to all the words in a set of predefined dictionaries and takes an average of all the sentiments in a sentence to generate the final valence score. Studies have suggested that positive emotions are significantly related to retweets [61].

TextBlob is a Python library for processing textual data. It provides a simple API for examining common NLP tasks, such as part-of-speech tagging, noun phrase extraction, sentiment analysis, classification, and translation. To extract emotional valence (polarity) and emotional intensity (subjectivity) data, we processed the data set pooled from the final analysis corpus of each vaccine stance. Initially, we used the Python function *NeatText*, a simple NLP package for cleaning textual data and text preprocessing; we removed user handlers, Universal Resource Locators (URLs), punctuation, non–American Standard Code for Information Interchange characters, numbers, hypertext markup language (html) tags, stopwords, special characters, emojis, and multiple spaces. We then used TextBlob to calculate the value of emotional valence and emotional intensity. Generally, the data are supplied as a bag-of-words, and after assigning individual scores to each word, the final sentiment is represented through a sum pooling of all the sentiments. TextBlob has semantic labels that facilitate fine-grained sentiment analysis. The workflow for calculating emotional valence and emotional intensity is presented in Multimedia Appendix 2.

#### Concreteness

Concreteness is an aspect of communication in which the information provided in a message is highly descriptive, specific, and vivid; users generally rely more on concrete wording to make their decisions [58]. Studies have suggested that individuals recall concrete words more effectively than abstract words [62] and that concrete words are more persuasive in affecting user behavior [61]; thus, we expected language concreteness to play a role in users’ decision to disseminate vaccine stance tweets. Examples of concreteness are presented in Multimedia Appendix 1.

To measure the content concreteness of cleaned tweets, we relied on the R package *doc2concrete* [63], which uses a dictionary of 40,000 common English words and expressions [64]. The concrete score has a range of 0-5, where 0 is abstract and 5 is concrete. The validity and reliability of this dictionary have been confirmed in the medical setting [65] and in online reviews [66]. Furthermore, this dictionary includes words from the medical domain. For example, “virus” has a concreteness rating of 3.48, whereas “vaccination” has a concreteness rating of 4.24. We calculated concreteness through the workflow presented in Multimedia Appendix 2.

### Predictors: Peripheral Route

#### Informational Social Influence

We measured informational social influence by using the “favorite” count (ie, number of likes) of a tweet. Researchers have studied informational social influence under the bandwagon effect and related concepts, such as herd behavior and social proof [45]. In practice, we took the square root of the favorite count to resolve convergence problems caused by its large scale (from 0 to nearly 30,000) in regression analysis. This approach has been used by researchers to normalize a skewed distribution. The resultant scale for the favorite count was from 0 to 173.1 for provaccine and from 0 to 100.5 for antivaccine tweets. We also used other normalization techniques, including the *z* score and min-max normalization; however, for the current model, these methods performed less well in the iterative procedure of maximum likelihood estimation.

#### Source Trustworthiness

The trustworthiness of a tweet is determined by whether the tweet is from a user whose status has been verified [56]. Twitter uses an authentication mechanism to ensure the authenticity of user identity, and a verified user is signified by a blue tick next to the screen name. Therefore, this variable is binary, with 1 indicating a trustworthy user and 0 reflecting a nontrustworthy user. Researchers have noted that tweets from verified users disseminate more rapidly than those from nonverified users [67].

#### Source Attractiveness

A Twitter user can follow any other user, and the number of followers reflects the likeability of the user’s real-world status. We measured source attractiveness as the number of followers. Studies that have utilized source attractiveness have identified a substantial effect of a user’s number of followers on the retweetability of a tweet [39,56]. We log-transformed the variable to render its scale comparable to other predictors.

### Regression Analysis and Sensitivity Analysis

We performed logistic regression and generalized negative binomial (NB) regression on the binary retweet outcome and the number of retweets, respectively. The generalized NB extends the NB mean dispersion model by providing flexibility in parameterizing the dispersion parameter α. We specified that the log of α is a linear function of the same covariates used in the main model. The chi-squared test rejected the null hypothesis that none of the covariates in the dispersion function have predictive power (*P*<.001). Akaike and Bayesian information criteria also indicated that the generalized NB is preferable to the NB model (Multimedia Appendix 3). The user-clustered sandwich variance estimator, which accommodates intragroup correlation of observations, was used to improve statistical inferences about regression coefficients. Because vaccine stance tweets were posted across multiple points in time, accounting for various exposures in generalized NB regressions was necessary. We included the log-transformed exposure variable, defined as the number of days from the tweet date to the last day of the study period, August 26, 2021. The correlation coefficient matrix (Multimedia Appendix 4) indicated that the correlation between predictors were generally low, except for the 3 peripheral-route variables, which were moderately correlated (0.3-0.4). All regressions were performed using Stata 16 software (Stata Corp Inc.).

We conducted sensitivity analyses to verify whether the results were robust for various model specifications. First, to capture common trends that may affect decisions of retweets, we included monthly binary variables for June, July, and August in logistic regression models. Data for April and May were combined to serve as the reference group; the results are summarized in Multimedia Appendix 5. Second, to avoid results being driven by outliers, we excluded tweets that had an exceptionally high number of retweets, using the top 0.5% as a cut-off point. As a result, provaccine tweets that had more than 83 retweets and antivaccine tweets with more than 325 retweets were excluded; see the results in Multimedia Appendix 6. All analyses revealed that our regression results remained consistent across various model specifications.

### Ethical Considerations

Informed consent cannot be obtained to analyze Twitter postings as Twitter posts are publicly available information.

## Results

### Summary Statistics

The summary statistics of the model variables are presented in Table 1. For provaccine and antivaccine tweets, 28% and 32% were retweeted and the average number of retweets was 3.16 and 8.86, respectively. These findings are consistent with the existing evidence that antivaxxers are more active in message sharing on Twitter [20]. The average number of hashtags was higher for antivaccine (3.18, SD 2.83) than for provaccine (2.82, SD 2.50) tweets. The mean emotional valence score was 0.07 (SD 0.30) for provaccine and 0.03 (SD 0.28) for antivaccine tweets, indicating that provaccine tweets had more positive emotions than antivaccine tweets. The mean emotional intensity score was similar (0.37, SD 0.34, and 0.35, SD 0.33) for the 2 groups. The mean concreteness score was 2.12 (SD 0.68) for provaccine and 1.92 (SD 0.66) for antivaccine tweets. The mean square root of the number of “likes” was 1.55 (SD 3.41) for provaccine and 1.76 (SD 4.56) for antivaccine tweets. Approximately 6% and 1% of provaccine and antivaccine messages, respectively, were tweeted by a verified user, which was considerably low. This finding accords with research that antivaccine messages are led by nonverified Twitter users [68]. The mean log number of followers was 6.82 (SD 2.06) for provaccine and 5.94 (SD 1.98) for antivaccine tweets.

**Table 1:** Summary of provaccine and antivaccine model variables.

Model variables			Provaccine tweets (N=141,782)						Antivaccine tweets (N=8556)				
			Mean (SD)		Minimum		Maximum	Mean (SD)			Minimum		Maximum
**Outcome variable**													
	Whether retweeted (0/1)	0.28 (0.45)		0		1		0.32 (0.47)		0		1	
	Retweet count	3.16 (60.87)		0		12,500		8.86 (99.72)		0		5141	
**Central route**													
	Number of hashtags	2.82 (2.50)		1		32		3.18 (2.83)		1		35	
	Number of mentions	0.72 (1.47)		0		24		0.70 (1.23)		0		14	
	Emotional valence score (–1 to 1)	0.07 (0.30)		–1		1		0.03 (0.28)		–1		1	
	Emotional intensity score (0–1)	0.37 (0.34)		0		1		0.35 (0.33)		0		1	
	Concreteness score (0–5)	2.12 (0.68)		0		4.59		1.92 (0.66)		0		3.74	
**Peripheral route**													
	Informational social influence: number of likes (square root)	1.55 (3.41)		0		173.12		1.76 (4.56)		0		100.5	
	Source trustworthiness: a verified user (0/1)	0.06 (0.24)		0		1		0.01 (0.12)		0		1	
	Source attractiveness: number of followers (log)	6.82 (2.06)		0		16.55		5.94 (1.98)		0		12.83	
	Exposure^a^ (log days)	3.20 (1.01)		0		4.81		3.39 (1.16)		0		4.81	

^a^Exposure is defined as the number of days from the tweet date to the last day of the study period, August 26, 2021.

### Central-Route Predictors

**Figure 3:**
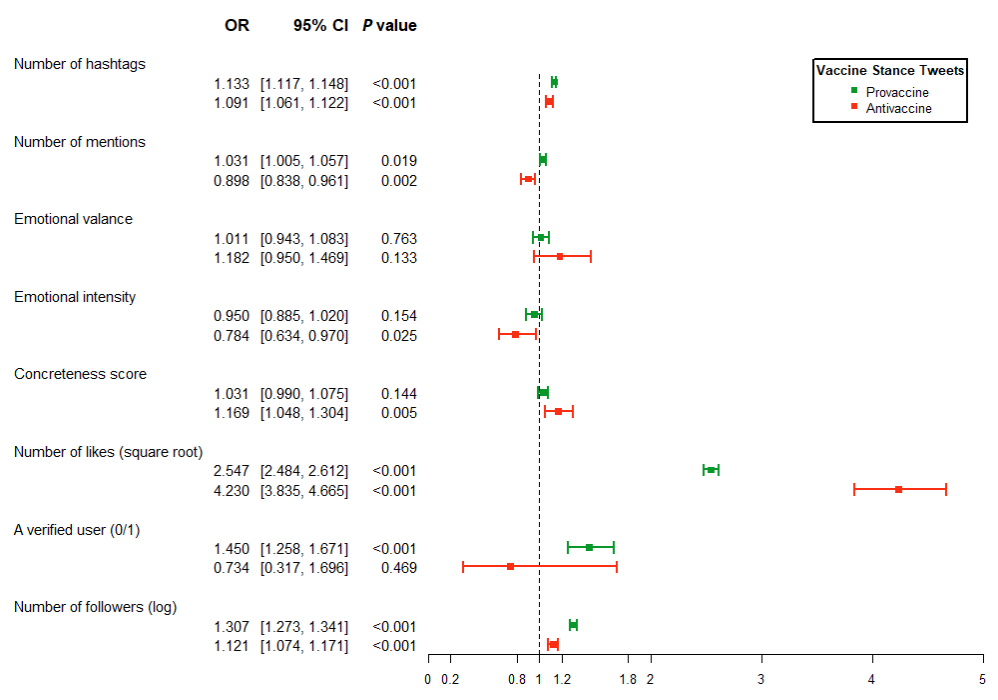
Results from logistic regressions of whether a vaccine stance message was retweeted. This figure illustrates the estimated OR associated with different characteristics of vaccine stance messages. The green color refers to provaccine tweets (N=141,782), and the red color refers to antivaccine tweets (N=8556). The horizontal line represents the 95% CI; the dot in the middle represents the estimate of the coefficient. The user-clustered sandwich variance estimator was used. OR: odds ratios.

**Figure 4:**
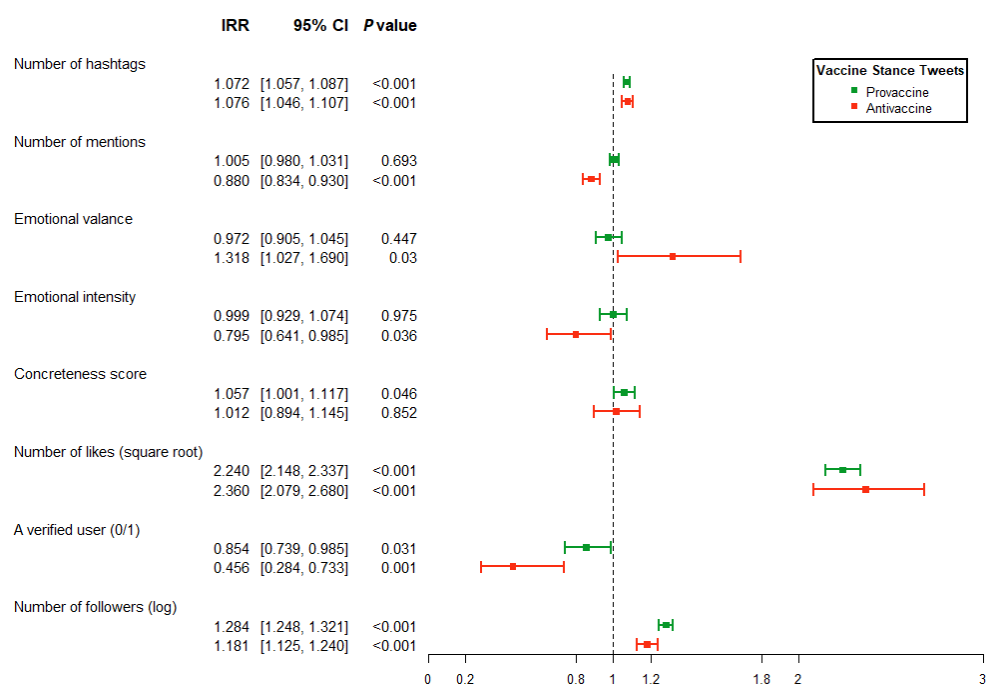
Results from generalized negative binomial regressions of the retweet count. This figure illustrates the estimated IRRs associated with different characteristics of vaccine stance messages. The green color refers to provaccine tweets (N=141,782), and the red color refers to antivaccine tweets (N=8556). The horizontal line represents the 95% CI; the dot in the middle represents the estimate of the coefficient. The user-clustered sandwich variance estimator was used. Exposure was included in the model with the coefficient constrained to 1. IRR: incidence rate ratio.

The results from the logistic and generalized NB regressions are presented in Figures 3 and 4, respectively. All regressions were run separately for provaccine (green color) and antivaccine (red color) tweets to examine the characteristics of messages that may determine the likelihood and number of retweets.

An additional hashtag increased the odds of sharing by 13.3% (95% CI 1.12-1.15, *P<*.001) and 9.1% (95% CI 1.06-1.12, *P*<.001) for provaccine and antivaccine tweets, respectively. An additional mention (of another user) increased the odds of sharing provaccine tweets by 3.1% (95% CI 1.01-1.06, *P=*.02) but reduced the odds of retweeting antivaccine tweets by 10.2% (95% CI 0.84-0.96, *P=*.002). A 1-point increase in the emotional intensity (subjectivity) score reduced the odds of sharing an antivaccine tweet substantially by 21.6% (95% CI 0.63-0.97, *P=*.03). Finally, a 1-point increase in concreteness scores increased the odds of sharing an antivaccine tweet substantially by 16.9% (95% CI 1.05-1.30, *P=*.01).

When the outcome variable was the number of retweets, we obtained similar results to those of the likelihood of retweets. The number of hashtags increased the retweet rate for both provaccine (incidence rate ratio [IRR]=1.07, 95% CI 1.06-1.09, *P<*.001) and antivaccine (IRR=1.08, 95% CI 1.05-1.11, *P<*.001) tweets. For antivaccine tweets, the number of mentions decreased the retweet rate by 12% (IRR=0.88, 95% CI 0.83-0.93, *P<*.001); positive valence increased the retweet rate substantially by 31.8% (IRR=1.32, 95% CI 1.03-1.69, *P=*.03), and emotional intensity decreased the retweet rate substantially by 20.5% (IRR=0.80, 95% CI 0.64-0.99, *P=*.04). With respect to provaccine tweets, a 1-point increase in the concreteness score increased the incidence rate of retweets marginally (IRR=1.06, 95% CI 1.00-1.12, *P=*.046).

### Peripheral-Route Predictors

The results associated with peripheral routes are presented in Figures 3 and 4 for the likelihood and number of retweets, respectively. An additional square-root number of likes increased the odds of retweets for provaccine and antivaccine messages by a factor of 2.55 (95% CI 2.48-2.61, *P<*.001) and 4.23 (95% CI 3.84-4.67, *P<*.001), respectively. Verification of user status increased the odds of retweets for provaccine messages substantially by 45% (95% CI 1.26-1.67, *P<*.001). A 1% increase in the number of followers increased the odds of retweeting provaccine and antivaccine messages by 30.7% (95% CI 1.27-1.34, *P<*.001) and 12.1% (95% CI 1.07-1.17, *P<*.001), respectively.

The generalized NB model indicated that provaccine and antivaccine tweets that had 1 more square-root number of likes had 2.24 (95% CI 2.15-2.34, *P<*.001) and 2.36 (95% CI 2.08-2.68, *P<*.001) times more retweets, respectively. When the author was a verified user, the rate of retweeting decreased for both groups (IRR=0.85 [pro], IRR=0.46 [anti], *P=*.03 [pro], *P=*.001 [anti]). In contrast, the number of followers increased the incidence rate of retweeting for both groups (IRR=1.28 [pro], IRR=1.18 [anti], *P<*.001).

## Discussion

### Principal Findings

This study applied the ELM to investigate characteristics of COVID-19 vaccine stance–related tweets that were associated with the likelihood and number of retweets on Twitter. The key finding is that content-related (central-route) predictors are strongly associated with retweets of antivaccine messages. Specifically, for antivaccine messages, the number of hashtags was positively associated with (the likelihood and number of) retweets; positive valence was associated with a higher number of retweets, concreteness was positively associated with the likelihood of retweets, whereas the number of mentions and emotional intensity were negatively associated with (the likelihood and number of) retweets. Regarding provaccine messages, only the number of hashtags was strongly and positively associated with (the likelihood and number of) retweets; the number of mentions and concreteness were positively but weakly associated with the likelihood of retweets. Among the content-unrelated (peripheral-route) predictors, the number of likes and followers were strongly and positively associated with (the likelihood and number of) retweets of provaccine and antivaccine messages.

### Central-Route Predictors Predominantly Associated with Dissemination of Antivaccine Tweets

The ELM predicts that if recipients have a high desire or ability to process a message, they will use the central route and spend more time deliberating on their decision. In this context, if antivaccine messages were mostly shared by antivaxxers, our finding of strong associations between central-route predictors and dissemination of antivaccine messages may imply that antivaxxers have relied more on cognitive cues than provaxxers to make retweeting decisions. Particularly, having positive emotions, low emotional intensity (objective content), and concrete words considerably increased the dissemination of antivaccine tweets. These results warrant attention because they conflict with the general perception that antivaxxers are irrational and attracted by negative emotions and abstract slogans [69-71].

The positive associations between concreteness and dissemination of antivaccine messages may be explained by the strategy used by antivaccine message creators to specify the harm caused by COVID-19 vaccines. Specifically, if antivaccine messages include concrete words, then it is likely to motivate the reader to share a descriptive, specific, and factual vaccine stance message. However, the same cannot be said when it comes to utilizing concrete words in provaccine messages, where there is little impact on readers' sharing of vaccine stance messages in this study.

The information systems literature contains inconsistent findings on valence (positive and negative) in electronic word-of-mouth studies [39,42,56]. A study demonstrated that negative valence has more influence on sharing online reviews of consumer products than positive valence [38]. Our work provides additional evidence that emotional valence predominantly has positive effects on retweeting antivaccine messages. Furthermore, the negative association between emotional intensity (subjectivity) and dissemination of antivaccine messages supports the existing research [39] that also indicates a negative effect of emotional intensity on the sharing of information behavior.

With respect to information richness, we discovered that hashtags increase the dissemination of both provaccine and antivaccine tweets, which is consistent with the findings of prior research [39]. Mentioning another user had a small negative effect on the dissemination of antivaccine messages, which is consistent with results that indicate mentions have a negative effect on information sharing [39]. One possible explanation for this is that in antivaccine messages, mentions are used to cite provaccine users, which is not welcomed by the antivaxxer community.

### Peripheral-Route Predictors Associated with Dissemination of Both Provaccine and Antivaccine Tweets

The number of likes (favorite count) measures social influence. It consistently demonstrated a positive association with dissemination of vaccine stance tweets in all models. The finding can be explained by the bandwagon effect, where people follow a trend regardless of the underlying evidence. This trend was stronger for antivaccine users than for provaccine users probably because of their desire to fit into the antivaxxers’ groups [72]. Existing research has revealed that a strong sense of community is a key factor contributing to the success of the antivaccination movement [20].

In the provaccine models, the association between the verified user status and retweets was inconsistent; in the antivaccine models, the verified user status was negatively associated with the number of retweets. This contradicts our hypothesis that tweets from verified users are more likely to be retweeted. One possible explanation for this trend is that the percentage of verified users was low in both groups (6% and 1% in the provaccine and antivaccine groups, respectively). The predictor varied little, which made fitting the regression line difficult. Moreover, the data revealed that the verified users received more likes and had more followers compared to the nonverified users; the 3 variables were correlated (correlation coefficients=0.3-0.4). When we excluded either the favorite count or the number of followers, the verified user status was positively associated with retweets in all models for provaccine tweets and in 1 model for antivaccine tweets (Multimedia Appendix 7).

Source attractiveness (number of followers) had positive associations with disseminating both provaccine and antivaccine messages. The literature indicates that having many followers leads to a higher probability of information dissemination [39].

### Recommendations for COVID-19 Vaccination Campaigns Using Social Media

This study provides several insights into how COVID-19 vaccination campaigns can be strengthened. First, to promote the dissemination of provaccine messages, policymakers may consider focusing on peripheral-route predictors (content-unrelated characteristics), such as increasing the likeability of their tweets, engaging with provaxxers who have many followers, and gaining more followers on Twitter. Moreover, to leverage central-route predictors, policymakers may use more hashtags in their messages. Using concrete words in a provaccine message may also increase the number of retweets the message receives, although the effect of doing so was small in this study.

Second, because antivaccine tweets with positive emotions, objective content, and concrete words are more likely to be disseminated, policymakers should pay attention to antivaccine messages with such characteristics. Additionally, paying attention to antivaccine tweets with many likes and followers could be crucial because those tweets are likely to be widely circulated. Research has demonstrated that dissemination of antivaccine messages is driven by strong influencers [20].

### Limitations

This study has several limitations. First, despite the popularity of Twitter, its users are a selected population and may not be representative of the general United States population. The identification of tweets may be incomplete because of a limited use of hashtags. Second, because Twitter has a strict policy of removing vaccine misinformation tweets from its platform, our data set may have been limited. Third, we examined a user’s retweeting decision when confronting a particular tweet. We were not able to identify people who retweeted those vaccine stance messages, and thus we could not be sure of their vaccine stances. Although most people retweet messages that are consistent with their own principles, some may retweet information that contradicts their beliefs. This limitation has been discussed in another Twitter studies [9] and should be considered when interpreting the results. Fourth, this study adopted content analysis and did not incorporate the effects of images or emoticons. In a literature review, we found that researchers removed emojis during preprocessing and cleaning of vaccine message text data to study multiple topics in Twitter, such as online vaccination debates [73], childhood vaccination opinions [74], COVID-19 vaccine sentiment in the United States [75], and key themes and topics on COVID-19 vaccines [76]. On similar lines of the literature, we removed emojis from the Twitter text corpus to analyze our dissemination model. However, emojis can enrich our findings by providing useful information alongside text tweets. Future research may consider including emojis in empirical analysis. Finally, 1 study utilized the data from Twitter posts and compared the sentiment outcomes of TextBlob, VADER, and Word2Vec–bidirectional long short-term memory (Word2Vec-BiLSTM) models. The results showed that TextBlob provides fewer positive sentiments compared to Word2Vec-BiLSTM but provides more positive sentiments compared to VADER [60]. Despite the wide applications of TextBlob on Twitter data for sentiment analysis [77,78], using different tools to validate emotional valence will help confirm the main findings of this study.

### Conclusion

This study identified the characteristics of COVID-19 vaccine stance tweets that are associated with the likelihood and number of retweets. This was performed by applying the ELM and examining 2 psychological routes involved in retweet decisions. A major finding of this study is that the dissemination of antivaccine messages is strongly associated with characteristics related to message content (central-route processing), including emotional valence and intensity. However, message content exhibited a much weaker association with dissemination of provaccine messages. We discovered that dissemination of provaccine messages is predominately determined by content-unrelated characteristics, such as the numbers of likes and followers.

## Appendix

Multimedia Appendix 1 Examples of emotional valence, emotional intensity, and concreteness in pro- and antivaccine tweets.

Multimedia Appendix 2 Workflow for calculating emotional valence, emotional intensity, and concreteness.

Multimedia Appendix 3 Akaike information criterion (AIC) and Bayesian information criterion (BIC) for model selection.

Multimedia Appendix 4 Correlation coefficient matrices for variables in the pro- and the antivaccine model.

Multimedia Appendix 5 Logistic regressions of whether a message was retweeted, with month indicators included.

Multimedia Appendix 6 Excluding vaccine stance–related tweets that had an exceptionally high number of retweets, using the top 0.5% as a cut-off point.

Multimedia Appendix 7 Regression results from the pro- and antivaccine models, excluding either the number of likes or the number of followers.

## Abbreviations

API: application programming interface
BiLSTM: bidirectional long short-term memory
CLT: construal level theory
ELM: elaboration likelihood model
IRR: incidence rate ratio
NB: negative binomial
NLP: natural language processing
OR: odds ratio
