# Supplementary material for: The Association Between Dissemination and Characteristics of Pro-/Anti-COVID-19 Vaccine Messages on Twitter: Application of the Elaboration Likelihood Model
Source: JMIR Infodemiology. 2022 Jun 27;2(1):e37077. doi: 10.2196/37077 (PMC9239316; doi:10.2196/37077)
Supplement: Multimedia Appendix 1 [file infodemiology_v2i1e37077_app1.docx]

**Multimedia Appendix 1. Examples of emotional valence, emotional intensity, and concreteness in pro- and anti-vaccine tweets**

Table A1. Examples of emotional valence, emotional intensity, and concreteness in antivaccine and provaccine tweets

| Stance | Variable | Tweet example | TextBlob score |
| --- | --- | --- | --- |
| Antivaccine | Emotional valence  (from negative -1 to positive 1) | Damn your evil intentions. God will crush your administration and your master, Satan himself. Disgusting creeps. #NoVaccinePassports | -1 |
|  |  | This is outrageous. Conspiracy theory becomes reality again  #NoVaccinePassports | -1 |
|  |  | Promise the people that you will never ever introduce domestic vaccine passports. #NoVaccinePassports | 0 |
|  |  | People should choose what’s best for them and No one should pressure you either way #NoVaccinePassports | 1 |
|  |  | @goodfoodgal #NaturalImmUNity is Safer and Better!!!! No vaccine Needed with #God | 1 |
|  | Emotional intensity  (from objective 0 to subjective 1) | How many people who have had #COVID19 (have #NaturalImmunity) have died? | 0 |
|  |  | I have signed #Together Declaration with over 200 campaign leaders, business groups & professionals. Please join us in signing and sharing the declaration so we can all have our voices heard? #together? | 0 |
|  |  | Full and fair debate. Freedom to choose. #novaccinepassports | 0.9 |
|  |  | They want to forcefully vaccinate you! Are you seeing how far this has gone?? #NoVaccinePassports | 1 |
|  | Concreteness  (from abstract 0 to concrete 5) | #NoVaccinePassports #NoMasks we are done with all that BS | 0 |
|  |  | Couldn't agree more, we can't let this happen. #NoVaccinePassports #NoVaccinePassportsAnywhere | 1.2 |
|  |  | Read the list of esteemed doctors from the most prestigious universities & hospitals who signed this letter; are they only #antivaxx #conspiracytheorists, too!? | 2.9 |
|  |  | No, I had the #NaturalImmunity #vaccination. I ate Dirt as a Kid and licked #Honey right off the Tree. No #jab for me. I am one of the 99.06% #Survivor rate. | 3.7 |
| Provaccine | Emotional valence  (from negative -1 to positive 1) | Read it...not something to be trifled with. People who refuse a #vaccine are morons! And they could kill us all, #vaccinated or not! | -1 |
|  |  | Get the #Jab or get the boot! #idiots | -0.8 |
|  |  | Now, can we do all travelers on planes, trains, boats, and buses #vaccinate | 0 |
|  |  | Speaking for only myself as a teacher I would be so very happy not to have myself and students not having to wear masks #vaccinate | 0.8 |
|  |  | Sleeves up, wheels up!  So great to see the @OchsnerHealth team at MSY helping to #vaccinate the community – and the world!!! | 1 |
|  | Emotional intensity  (from objective 0 to subjective 1) | Get your kids 12-18 #vaccinated for school | 0 |
|  |  | #VaccinesWork Thanks for this Dr. Kwan! | 0.2 |
|  |  | Hot bath to soothe the aches post vaccine. #vaccinated | 0.8 |
|  |  | It's time to mask up again in schools. If only everyone had gotten #vaccinated we might not have to be doing this again. | 1 |
|  | Concreteness  (from abstract 0 to concrete 5) | Just gonna RT this. #GetVaccinated | 0 |
|  |  | To all my new followers and follow-ees, yes I am #completely #VAXXED (#Moderna) and am encouraging other people to to the same. | 1.9 |
|  |  | Proud to live in #Maryland where there is no longer a #vaccine shortage. We have one of the highest #vaccination rates. #GetVaccinated | 2.5 |
|  |  | Why 98% of Hospital Physicians get #vaccinated. Perhaps you should have listened. | 4.0 |
