# Supplementary material for: The Association Between Dissemination and Characteristics of Pro-/Anti-COVID-19 Vaccine Messages on Twitter: Application of the Elaboration Likelihood Model
Source: JMIR Infodemiology. 2022 Jun 27;2(1):e37077. doi: 10.2196/37077 (PMC9239316; doi:10.2196/37077)
Supplement: Multimedia Appendix 2 [file infodemiology_v2i1e37077_app2.docx]

**Multimedia Appendix 2. Workflow for calculating emotional valence, emotional intensity, and concreteness**


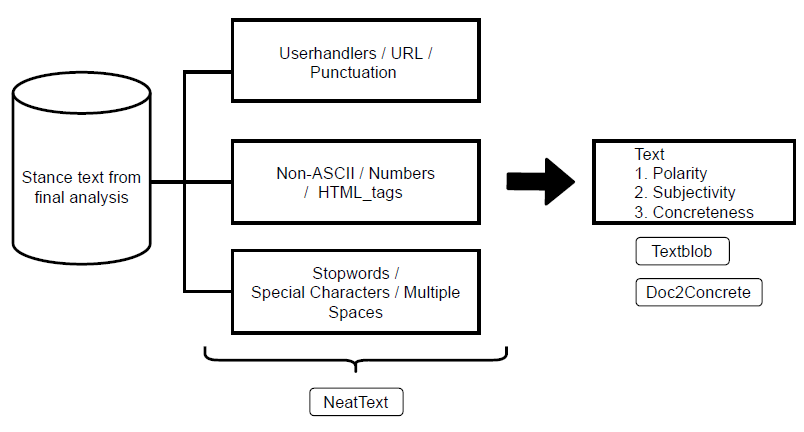


Figure A2. Workflow for calculating emotional valence, emotional intensity, and concreteness using NeatText (Python), TextBlob (Python), and doc2concrete (R package).
