# Supplementary material for: The Association Between Dissemination and Characteristics of Pro-/Anti-COVID-19 Vaccine Messages on Twitter: Application of the Elaboration Likelihood Model
Source: JMIR Infodemiology. 2022 Jun 27;2(1):e37077. doi: 10.2196/37077 (PMC9239316; doi:10.2196/37077)
Supplement: Multimedia Appendix 3 [file infodemiology_v2i1e37077_app3.docx]

**Multimedia Appendix 3. Akaike's information criterion (AIC) and Bayesian information criterion (BIC) for model selection**

Table A3.1 Akaike's information criterion (AIC) and Bayesian information criterion (BIC)

for provaccine models

| Model | N | Log-likelihood (null) | Log likelihood (model) | Degree of freedom | AIC | BIC |
| --- | --- | --- | --- | --- | --- | --- |
| Negative Binomial (NB) | 141 782 | -192 454.3 | -154 111.2 | 10 | 308 242.3 | 308 340.9 |
| Generalized NB | 141 782 | -184 998.8 | -150 669.7 | 18 | 301 375.4 | 301 553 |

Table A3.2 Akaike's information criterion (AIC) and Bayesian information criterion (BIC)

for antivaccine models

| Model | N | Log-likelihood (null) | Log likelihood (model) | Degree of freedom | AIC | BIC |
| --- | --- | --- | --- | --- | --- | --- |
| Negative Binomial (NB) | 8 556 | -13 918.08 | -11 222.52 | 10 | 22 465.05 | 22 535.59 |
| Generalized NB | 8 556 | -13 578.98 | -10 862 | 18 | 21 759.99 | 21 886.97 |
