# Supplementary material for: The Association Between Dissemination and Characteristics of Pro-/Anti-COVID-19 Vaccine Messages on Twitter: Application of the Elaboration Likelihood Model
Source: JMIR Infodemiology. 2022 Jun 27;2(1):e37077. doi: 10.2196/37077 (PMC9239316; doi:10.2196/37077)
Supplement: Multimedia Appendix 4 [file infodemiology_v2i1e37077_app4.docx]

**Multimedia Appendix 4 Correlation coefficient matrices for variables in the pro- and anti-vaccine model**

Table A4.1 Correlation coefficient matrix for variables in the provaccine model (N=141 782)

| Variable | | 1 | 2 | 3 | 4 | 5 | 6 | 7 | 8 | 9 | 10 |
| --- | --- | --- | --- | --- | --- | --- | --- | --- | --- | --- | --- |
| 1 | Whether retweeted (0/1) | 1.00 |  |  |  |  |  |  |  |  |  |
| 2 | Retweet count | 0.08 | 1.00 |  |  |  |  |  |  |  |  |
| 3 | Number of hashtags | 0.05 | -0.01 | 1.00 |  |  |  |  |  |  |  |
| 4 | Number of mentions | 0.01 | -0.01 | -0.03 | 1.00 |  |  |  |  |  |  |
| 5 | Emotional valence | 0.04 | 0.00 | 0.00 | 0.01 | 1.00 |  |  |  |  |  |
| 6 | Emotional intensity | 0.01 | 0.00 | -0.02 | -0.01 | 0.14 | 1.00 |  |  |  |  |
| 7 | Concreteness | 0.02 | 0.01 | -0.27 | -0.03 | 0.02 | 0.13 | 1.00 |  |  |  |
| 8 | Number of likes (square root) | 0.40 | 0.58 | -0.05 | -0.03 | 0.04 | 0.02 | 0.05 | 1.00 |  |  |
| 9 | A verified user (0/1) | 0.26 | 0.09 | -0.04 | -0.03 | 0.05 | 0.01 | 0.04 | 0.31 | 1.00 |  |
| 10 | Number of followers (log) | 0.38 | 0.09 | -0.06 | 0.01 | 0.03 | -0.01 | -0.01 | 0.34 | 0.39 | 1.00 |

Table A4.2 Correlation coefficient matrix for variables in the antivaccine model (N=8 556)

| Variable | | 1 | 2 | 3 | 4 | 5 | 6 | 7 | 8 | 9 | 10 |
| --- | --- | --- | --- | --- | --- | --- | --- | --- | --- | --- | --- |
| 1 | Whether retweeted (0/1) | 1.00 |  |  |  |  |  |  |  |  |  |
| 2 | Retweet count | 0.13 | 1.00 |  |  |  |  |  |  |  |  |
| 3 | Number of hashtags | 0.04 | -0.03 | 1.00 |  |  |  |  |  |  |  |
| 4 | Number of mentions | -0.08 | -0.04 | -0.08 | 1.00 |  |  |  |  |  |  |
| 5 | Emotional valence | 0.02 | 0.02 | 0.01 | 0.02 | 1.00 |  |  |  |  |  |
| 6 | Emotional intensity | -0.02 | 0.02 | -0.02 | 0.01 | 0.00 | 1.00 |  |  |  |  |
| 7 | Concreteness | 0.05 | 0.05 | -0.35 | -0.01 | -0.04 | 0.16 | 1.00 |  |  |  |
| 8 | Number of likes (square root) | 0.39 | 0.74 | -0.06 | -0.07 | 0.03 | 0.02 | 0.09 | 1.00 |  |  |
| 9 | A verified user (0/1) | 0.14 | 0.30 | -0.04 | -0.02 | 0.01 | 0.01 | 0.04 | 0.39 | 1.00 |  |
| 10 | Number of followers (log) | 0.33 | 0.19 | -0.02 | -0.05 | 0.02 | -0.01 | 0.03 | 0.40 | 0.27 | 1.00 |
