# Supplementary material for: The Association Between Dissemination and Characteristics of Pro-/Anti-COVID-19 Vaccine Messages on Twitter: Application of the Elaboration Likelihood Model
Source: JMIR Infodemiology. 2022 Jun 27;2(1):e37077. doi: 10.2196/37077 (PMC9239316; doi:10.2196/37077)
Supplement: Multimedia Appendix 5 [file infodemiology_v2i1e37077_app5.docx]

**Multimedia Appendix 5 Logistic regressions of whether a message was retweeted, with month indicators included**

Table A5.1: Results from logistic regressions of whether a vaccine stance message was retweeted, with indicators for June, July and August

| **Y: whether a provaccine message was retweeted (N=141 782)** | | | | |
| --- | --- | --- | --- | --- |
| Predictor | Odd ratio | 95% CI | *P* value |  |
| Number of hashtags | 1.136 | [ 1.120 , 1.153 ] | <.001 |  |
| Number of mentions | 1.031 | [ 1.005 , 1.057 ] | 0.017 |  |
| Emotional valence | 1.047 | [ 0.976 , 1.123 ] | 0.203 |  |
| Emotional intensity | 0.931 | [ 0.867 , 1.000 ] | 0.051 |  |
| Concreteness | 1.044 | [ 1.001 , 1.089 ] | 0.043 |  |
| Number of likes (square root) | 2.580 | [ 2.515 , 2.646 ] | <.001 |  |
| Whether a verified user (0/1) | 1.474 | [ 1.277 , 1.701 ] | <.001 |  |
| Number of followers (log) | 1.301 | [ 1.267 , 1.335 ] | <.001 |  |
| June | 1.340 | [ 1.217 , 1.476 ] | <.001 |  |
| July | 1.496 | [ 1.368 , 1.636 ] | <.001 |  |
| August | 1.608 | [ 1.464 , 1.767 ] | <.001 |  |
|  |  |  |  |  |
| **Y: whether an antivaccine message was retweeted (N=8 556)** | | | | |
| Predictor | Odd ratio | 95% CI | *P* value |  |
| Number of hashtags | 1.090 | [ 1.06 , 1.121 ] | <.001 |  |
| Number of mentions | 0.903 | [ 0.842 , 0.968 ] | 0.004 |  |
| Emotional valence | 1.181 | [ 0.949 , 1.47 ] | 0.136 |  |
| Emotional intensity | 0.787 | [ 0.636 , 0.973 ] | 0.027 |  |
| Concreteness | 1.177 | [ 1.054 , 1.313 ] | 0.004 |  |
| Number of likes (square root) | 4.257 | [ 3.86 , 4.695 ] | <.001 |  |
| Whether a verified user (0/1) | 0.723 | [ 0.308 , 1.697 ] | 0.456 |  |
| Number of followers (log) | 1.121 | [ 1.074 , 1.171 ] | <.001 |  |
| June | 0.824 | [ 0.623 , 1.09 ] | 0.176 |  |
| July | 0.907 | [ 0.698 , 1.18 ] | 0.468 |  |
| August | 1.108 | [ 0.864 , 1.421 ] | 0.420 |  |

Note: For month indicators, April and May were combined to serve as the reference group. The user-clustered sandwich variance estimator was used.
