# Supplementary material for: The Association Between Dissemination and Characteristics of Pro-/Anti-COVID-19 Vaccine Messages on Twitter: Application of the Elaboration Likelihood Model
Source: JMIR Infodemiology. 2022 Jun 27;2(1):e37077. doi: 10.2196/37077 (PMC9239316; doi:10.2196/37077)
Supplement: Multimedia Appendix 6 [file infodemiology_v2i1e37077_app6.docx]

**Multimedia Appendix 6 Excluding vaccine stance-related tweets that had an exceptionally high number of retweets, using the top 0.5% as a cut-off point**

Table A6.1: Results from logistic regressions of whether a vaccine stance message was retweeted, excluding outliers

| **Y: whether a provaccine message was retweeted (N=141 073)** | | | | |
| --- | --- | --- | --- | --- |
| **Predictor** | **Odd ratio** | **95% CI** | **P value** |  |
| Number of hashtags | 1.133 | [ 1.117 , 1.148 ] | <.001 |  |
| Number of mentions | 1.031 | [ 1.005 , 1.057 ] | 0.019 |  |
| Emotional valence | 1.011 | [ 0.943 , 1.083 ] | 0.762 |  |
| Emotional intensity | 0.950 | [ 0.885 , 1.020 ] | 0.154 |  |
| Concreteness | 1.031 | [ 0.990 , 1.075 ] | 0.144 |  |
| Number of likes (square root) | 2.547 | [ 2.483 , 2.612 ] | <.001 |  |
| Whether a verified user (0/1) | 1.450 | [ 1.258 , 1.671 ] | <.001 |  |
| Number of followers (log) | 1.307 | [ 1.273 , 1.341 ] | <.001 |  |
| **Y: whether an antivaccine message was retweeted (N=8 513)** | | | | |
| **Predictor** | **Odd ratio** | **95% CI** | ***P* value** |  |
| Number of hashtags | 1.091 | [ 1.061 , 1.122 ] | <.001 |  |
| Number of mentions | 0.898 | [ 0.838 , 0.961 ] | 0.002 |  |
| Emotional valence | 1.182 | [ 0.950 , 1.469 ] | 0.133 |  |
| Emotional intensity | 0.784 | [ 0.634 , 0.970 ] | 0.025 |  |
| Concreteness | 1.169 | [ 1.048 , 1.304 ] | 0.005 |  |
| Number of likes (square root) | 4.230 | [ 3.835 , 4.665 ] | <.001 |  |
| Whether a verified user (0/1) | 0.734 | [ 0.317 , 1.696 ] | 0.469 |  |
| Number of followers (log) | 1.121 | [ 1.074 , 1.171 ] | <.001 |  |

Note: The user-clustered sandwich variance estimator was used.

Table A6.2: Results from generalized negative binomial regressions of retweet count, excluding outliers

| **Y: Number of retweets a provaccine message received (N=141 073)** | | | | |
| --- | --- | --- | --- | --- |
| **Predictor** | **IRR** | **95% CI** | **P value** |  |
| Number of hashtags | 1.074 | [ 1.059 , 1.089 ] | <.001 |  |
| Number of mentions | 1.003 | [ 0.978 , 1.028 ] | 0.825 |  |
| Emotional valence | 0.964 | [ 0.897 , 1.036 ] | 0.316 |  |
| Emotional intensity | 0.988 | [ 0.918 , 1.063 ] | 0.745 |  |
| Concreteness | 1.055 | [ 0.999 , 1.113 ] | 0.055 |  |
| Number of likes (square root) | 2.379 | [ 2.289 , 2.471 ] | <.001 |  |
| Whether a verified user (0/1) | 0.847 | [ 0.733 , 0.979 ] | 0.025 |  |
| Number of followers (log) | 1.273 | [ 1.237 , 1.309 ] | <.001 |  |
| **Y: Number of retweets an antivaccine message received (N=8 513)** | | | | |
| **Predictor** | **IRR** | **95% CI** | ***P* value** |  |
| Number of hashtags | 1.077 | [ 1.046 , 1.108 ] | <.001 |  |
| Number of mentions | 0.880 | [ 0.833 , 0.928 ] | <.001 |  |
| Emotional valence | 1.325 | [ 1.035 , 1.696 ] | 0.025 |  |
| Emotional intensity | 0.796 | [ 0.644 , 0.985 ] | 0.036 |  |
| Concreteness | 1.009 | [ 0.894 , 1.14 ] | 0.879 |  |
| Number of likes (square root) | 2.506 | [ 2.236 , 2.808 ] | <.001 |  |
| Whether a verified user (0/1) | 0.477 | [ 0.302 , 0.753 ] | 0.001 |  |
| Number of followers (log) | 1.167 | [ 1.113 , 1.224 ] | 0.000 |  |

Note: IRR=incidence rate ratio. The user-clustered sandwich variance estimator was used. Exposure was included in the model with coefficient constraint to 1.
