# Supplementary material for: The Association Between Dissemination and Characteristics of Pro-/Anti-COVID-19 Vaccine Messages on Twitter: Application of the Elaboration Likelihood Model
Source: JMIR Infodemiology. 2022 Jun 27;2(1):e37077. doi: 10.2196/37077 (PMC9239316; doi:10.2196/37077)
Supplement: Multimedia Appendix 7 [file infodemiology_v2i1e37077_app7.docx]

**Multimedia Appendix 7 Regression results from the pro- and anti-vaccine model, excluding either the number of likes or number of followers**

Table A7.1: Results from the provaccine model (N=141 782), excluding either the number of likes or number of followers

|  | **Logistic regression** | | | | | | |  | **Generalized negative binomial regression** | | | | | | |
| --- | --- | --- | --- | --- | --- | --- | --- | --- | --- | --- | --- | --- | --- | --- | --- |
|  | **Model I** | | |  | **Model II** | | |  | **Model III** | | |  | **Model IV** | | |
| **Predictor** | **OR** | **[95% conf. Int.]** | **P** |  | **OR** | **[95% conf. Int.]** | **P** |  | **IRR** | **[95% conf. int.]** | **P** |  | **IRR** | **[95% conf. int.]** | **P** |
| Number of likes (square root) |  |  |  |  | 2.764 | [ 2.694 , 2.836 ] | <.001 |  |  |  |  |  | 2.492 | [ 2.375 , 2.614 ] | <.001 |
| Number of followers (log) | 1.553 | [ 1.150 , 1.596 ] | <.001 |  |  |  |  |  | 1.845 | [ 1.762 , 1.931 ] | <.001 |  |  |  |  |
| Whether a verified user (0/1) | 2.624 | [ 2.265 , 3.039 ] | <.001 |  | 2.654 | [ 2.310 , 3.049 ] | <.001 |  | 1.550 | [ 1.207 , 1.991 ] | 0.001 |  | 1.422 | [ 1.214 , 1.664 ] | <.001 |

Note: OR=odds ratio. IRR=incidence rate ratio. The model included all central route predictors. The user-clustered sandwich variance estimator was used. Exposure was included in the generalized negative binomial model with coefficient constraint to 1.

Table A7.2: Results from the antivaccine model (N=8 556), excluding either the number of likes or number of followers

|  | | **Logistic regression** | | | | | | |  | | **Generalized negative binomial regression** | | | | | | | | |
| --- | --- | --- | --- | --- | --- | --- | --- | --- | --- | --- | --- | --- | --- | --- | --- | --- | --- | --- | --- |
|  | **Model V** | | | |  | **Model VI** | | | |  | | **Model VII** | | |  | **Model VIII** | | |  |
| **Predictor** | **IRR** | | **[95% conf. Int.]** | **P** |  | **IRR** | **[95% conf. Int.]** | **P** | |  | | **IRR** | **[95% conf. int.]** | **P** |  | **IRR** | **[95% conf. int.]** | **P** |  |
| Number of likes (square root) |  | |  |  |  | 4.420 | [ 4.015 , 4.865 ] | <.001 | |  | |  |  |  |  | 2.566 | [ 2.275 , 2.894 ] | <.001 |  |
| Number of followers (log) | 1.484 | | [ 1.400 , 1.573 ] | <.001 |  |  |  |  | |  | | 2.054 | [ 1.749 , 2.411 ] | <.001 |  |  |  |  |  |
| Whether a verified user (0/1) | 2.515 | | [ 1.453 , 4.355 ] | 0.001 |  | 1.033 | [ 0.441 , 2.418 ] | 0.941 | |  | | 3.140 | [ 0.823 , 11.997 ] | 0.094 |  | 0.746 | [ 0.484 , 1.151 ] | 0.185 |  |

Note: OR=odds ratio. IRR=incidence rate ratio. The model included all central route predictors. The user-clustered sandwich variance estimator was used. Exposure was included in the generalized negative binomial model with coefficient constraint to 1.
